# Missing Teeth and Incident Cardiovascular Disease in Ageing Populations: Longitudinal Evidence from Three Nationally Representative Cohorts

**DOI:** 10.3390/jcm15156080

**Published:** 2026-08-05

**Authors:** Zeping Huang, En Qiao, Jun Yu, Wei Wang

**Affiliations:** 1 Department of Structural Heart Disease, Fuwai Hospital, National Center for Cardiovascular Disease, Chinese Academy of Medical Sciences and Peking Union Medical College, Beijing 100037, China; hzp_bruce@126.com; 2 Department of Lung Transplantation and Thoracic Surgery, The First Affiliated Hospital, Zhejiang University School of Medicine, Hangzhou 310010, China; drqiaoen123@163.com; 3 Department of Thoracic Surgery, Fujian Medical University Union Hospital, Fuzhou 350001, China; egbert-yujun@outlook.com

**Keywords:** missing teeth, cardiovascular disease, oral health, ageing, CHARLS, HRS, ELSA, longitudinal cohort

## Abstract

**Background:** Cardiovascular disease (CVD) prevention in ageing populations requires simple and clinically accessible markers that may help identify individuals at elevated risk. Tooth loss is a common oral condition in middle-aged and older adults and may reflect cumulative inflammatory burden, impaired oral function, and broader health vulnerability. However, longitudinal evidence from nationally representative populations across different countries remains limited. We investigated whether a baseline of missing teeth was independently associated with incident CVD in three nationally representative ageing cohorts from China, the United States, and England. **Methods:** We analyzed data from the China Health and Retirement Longitudinal Study (CHARLS), the Health and Retirement Study (HRS), and the English Longitudinal Study of Ageing (ELSA). Participants aged 45 years or older without baseline CVD and with available baseline tooth status were included. Missing teeth were assessed at baseline as a dichotomous exposure. Incident CVD was defined as newly reported heart disease and/or stroke during follow-up. Cox proportional hazards models were used to estimate hazard ratios (HRs) and 95% confidence intervals (CIs), with sequential adjustment for sociodemographic, lifestyle, and clinical covariates. Kaplan–Meier, subgroup, and sensitivity analyses were also performed. **Results:** A total of 31,805 participants were included, comprising 13,451 from CHARLS, 11,384 from HRS, and 6970 from ELSA. During follow-up, 2336 incident CVD events occurred in CHARLS, 2914 in HRS, and 545 in ELSA. In fully adjusted models, missing teeth was associated with a higher risk of incident CVD in all three cohorts, with HRs of 1.31 (95% CI, 1.14–1.50) in CHARLS, 1.43 (95% CI, 1.29–1.57) in HRS, and 1.86 (95% CI, 1.49–2.31) in ELSA. Kaplan–Meier analyses showed consistently lower CVD-free survival among participants with missing teeth, and the associations remained broadly robust across subgroup and sensitivity analyses. **Conclusions:** In three nationally representative cohorts from China, the United States, and England, a baseline of missing teeth was independently associated with a higher risk of incident CVD among middle-aged and older adults. These findings suggest that missing teeth may serve as a simple and clinically accessible marker of elevated cardiovascular risk and support greater integration of oral health into cardiovascular risk assessment and preventive strategies for ageing populations.

## 1. Introduction

Cardiovascular disease (CVD) remains the leading cause of death and disability worldwide, and its burden continues to rise with population ageing and the accumulation of cardiometabolic risk factors [1]. In parallel, oral conditions remain among the most prevalent non-communicable diseases globally, with severe periodontitis and total tooth loss continuing to contribute substantially to disability and impaired quality of life [2]. Given the increasing prevalence of both CVD and tooth loss with advancing age, clarifying their relationship may have important implications for risk assessment and prevention in middle-aged and older adults.

Tooth loss is often the cumulative consequence of chronic oral diseases, particularly periodontitis and dental caries, and may also reflect long-term exposure to adverse health behaviors, limited dental care, and socioeconomic disadvantage. Several biological and behavioral pathways may plausibly link tooth loss to CVD. Chronic periodontal inflammation may promote systemic inflammatory activation, endothelial dysfunction, and atherogenesis [3,4,5]. In addition, tooth loss may impair mastication and alter dietary intake, favoring lower consumption of fruits, vegetables, and other cardioprotective foods [6]. Therefore, missing teeth may represent not only a marker of poor oral health but also a clinically accessible indicator of broader systemic vulnerability.

Previous epidemiological studies have increasingly suggested a positive association between tooth loss and cardiovascular outcomes, although the magnitude and interpretation of this relationship remain debated. A dose–response meta-analysis of prospective cohort studies reported that tooth loss was associated with increased risks of CVD and stroke [7]. In a national Scottish cohort, edentulousness was associated with higher CVD mortality, particularly stroke mortality [8]. Similarly, a very large Korean cohort study found that poorer oral health, including tooth loss, was associated with later coronary heart disease [9]. Additional meta-analytic evidence has linked tooth loss to mortality outcomes and to atherosclerotic cardiovascular disease-related events, although some analyses have highlighted heterogeneity and the possibility of residual confounding [10,11].

Despite this growing body of evidence, most previous studies have been conducted in single-country settings, which may limit generalizability. Whether the association between missing teeth and incident CVD is reproducible across populations with different healthcare systems, social environments, and oral health profiles remains insufficiently understood. The China Health and Retirement Longitudinal Study (CHARLS), the Health and Retirement Study (HRS), and the English Longitudinal Study of Ageing (ELSA) are three nationally representative longitudinal ageing cohorts from China, the United States, and England, respectively [12,13,14]. These datasets provide an important opportunity to examine the consistency of the association between missing teeth and incident CVD across diverse settings using a harmonized analytic framework.

Therefore, in the present study, we examined whether a baseline of missing teeth was independently associated with incident cardiovascular disease in three nationally representative ageing cohorts from China, the United States, and England. By applying a harmonized longitudinal analytic framework to the China Health and Retirement Longitudinal Study (CHARLS), the Health and Retirement Study (HRS), and the English Longitudinal Study of Ageing (ELSA), we aimed to determine whether the association was consistent across populations with different healthcare systems, social environments, and oral health profiles. We further assessed the robustness of this association across major demographic and clinical subgroups and in a series of sensitivity analyses. We hypothesized that missing teeth would be associated with a higher risk of subsequent CVD and could serve as a simple, clinically accessible marker relevant to cardiovascular risk stratification in middle-aged and older adults.

## 2. Materials and Methods

### 2.1. Study Design and Data Sources

This study was a longitudinal analysis based on three nationally representative ageing cohorts: CHARLS, HRS, and ELSA [12,13,14]. CHARLS is a nationally representative longitudinal survey of Chinese adults aged 45 years or older and their spouses [12]. HRS is a nationally representative longitudinal study of older adults in the United States [13]. ELSA is a panel study of community-dwelling adults aged 50 years or older in England [14].

**Figure 1:**
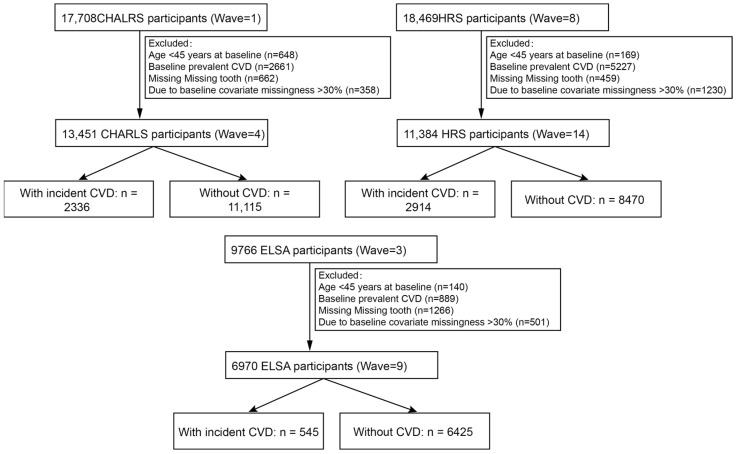
Flowchart of participant selection in the CHARLS, HRS, and ELSA cohorts. Initial samples included 17,708 participants from CHARLS (Wave 1), 18,469 from HRS (Wave 8), and 9766 from ELSA (Wave 3). Participants were excluded if they were younger than 45 years at baseline, had prevalent cardiovascular disease (CVD), had missing baseline information on tooth status, or had more than 30% missingness in key baseline covariates. The final analytic sample consisted of 13,451 participants in CHARLS, 11,384 in HRS, and 6970 in ELSA. CHARLS, China Health and Retirement Longitudinal Study; HRS, Health and Retirement Study; ELSA, English Longitudinal Study of Ageing; CVD, cardiovascular disease.

For the present analysis, baseline data were obtained from CHARLS Wave 1, HRS Wave 8, and ELSA Wave 3. Participants were excluded if they were younger than 45 years at baseline, had prevalent CVD, had missing information on baseline tooth status, or had more than 30% missingness in key baseline covariates. The participant selection process is shown in Figure 1.

### 2.2. Exposure Assessment

The primary exposure was baseline missing teeth status. In each cohort, tooth status was obtained from baseline questionnaire data and harmonized as a binary variable indicating the presence or absence of missing teeth. To maximize comparability across CHARLS, HRS, and ELSA, the exposure was analyzed dichotomously as “yes” versus “no”. Because ELSA provided information on the number of missing teeth, an additional ELSA-specific sensitivity analysis was conducted to examine whether the association differed according to the severity of tooth loss.

### 2.3. Outcome Assessment

The primary outcome was incident CVD during follow-up, defined as the first report of physician-diagnosed heart disease and/or stroke. The composite outcome was harmonized across CHARLS, HRS, and ELSA to improve comparability. We recognize that the exact wording and clinical scope of self-reported heart disease may differ somewhat across cohorts according to the original survey instruments. Follow-up time was calculated from baseline to the first occurrence of incident CVD, death, loss to follow-up, or the end of follow-up, whichever came first.

### 2.4. Covariates

Potential confounders were selected a priori on the basis of previous literature examining oral health and cardiovascular risk [3,4,5,6,7,8,9,10,11]. These included age, sex, education level, marital status, body mass index (BMI), smoking status, drinking status, hypertension, diabetes mellitus, cancer, chronic lung disease, and arthritis. Residence (urban/rural) was additionally described where available. Baseline characteristics of study participants are presented in Table 1.

### 2.5. Statistical Analysis

Continuous variables were summarized as medians with interquartile ranges (IQRs), and categorical variables were summarized as counts with percentages. Baseline characteristics were compared across cohorts using the Kruskal–Wallis test for continuous variables and the chi-square test for categorical variables. Standardized mean differences (SMDs) were additionally calculated to quantify the magnitude of between-cohort differences.

CVD-free survival according to baseline missing teeth status was evaluated using Kaplan–Meier curves and compared using the log-rank test (Figure 3) [15]. Associations between baseline missing teeth and incident CVD were estimated in each cohort using Cox proportional hazards regression models (Figure 4) [16]. Three sequential models were fitted. Model 1 was unadjusted. Model 2 was adjusted for age, sex, education level, and marital status. Model 3 was further adjusted for BMI, smoking, drinking, hypertension, diabetes mellitus, cancer, chronic lung disease, and arthritis. Hazard ratios (HRs) and 95% confidence intervals (CIs) were reported.

Subgroup analyses were conducted using the fully adjusted model across prespecified strata of age, sex, education level, smoking status, diabetes mellitus, and BMI (Figure 5). To avoid overadjustment and collinearity, the stratification variable was excluded from the corresponding subgroup model. Multiplicative interactions were assessed by likelihood ratio tests comparing models with and without the interaction term.

Several sensitivity analyses were performed to assess the robustness of the main findings (Figure 6). These analyses included restricting the outcome to heart disease only, restricting the outcome to stroke only, excluding events occurring within the first two years of follow-up, complete-case analysis, and competing-risk analysis using the Fine–Gray subdistribution hazards model [17]. The proportional hazards assumption was evaluated using Schoenfeld residual-based methods [18,19]. All analyses were performed using R software (version 4.3.2; R Foundation for Statistical Computing, Vienna, Austria), and a two-sided *p* value < 0.05 was considered statistically significant.

## 3. Results

### 3.1. Study Population and Baseline Characteristics

The participant selection process is shown in Figure 1. After excluding individuals who were younger than 45 years at baseline, had prevalent cardiovascular disease (CVD), had missing information on baseline tooth status, or had more than 30% missingness in key baseline covariates, a total of 31,805 participants were included in the final analysis, comprising 13,451 from CHARLS, 11,384 from HRS, and 6970 from ELSA. During follow-up, 2336 incident CVD events were identified in CHARLS, 2914 in HRS, and 545 in ELSA.

Baseline characteristics are summarized in Table 1. The median age was 57.0 years in CHARLS, 65.0 years in HRS, and 62.0 years in ELSA. Women accounted for 50.5% of participants in CHARLS, 62.2% in HRS, and 57.3% in ELSA. The prevalence of baseline missing teeth differed across cohorts, being 8.2% in CHARLS, 14.7% in HRS, and 13.0% in ELSA. Significant between-cohort differences were also observed for education level, marital status, BMI, smoking, drinking, hypertension, diabetes mellitus, cancer, chronic lung disease, and arthritis (all *p* < 0.001). The three cohorts differed substantially in demographic, behavioral, and clinical characteristics. CHARLS participants were younger and had lower BMI, whereas HRS and ELSA participants had higher BMI and different cardiometabolic profiles. Differences were also observed in smoking, drinking, hypertension, diabetes mellitus, chronic diseases, and baseline missing teeth. These findings indicate that the three cohorts represented distinct ageing populations from different social and healthcare contexts.

### 3.2. Distribution of Baseline Missing Teeth Across Sociodemographic Subgroups

**Figure 2:**
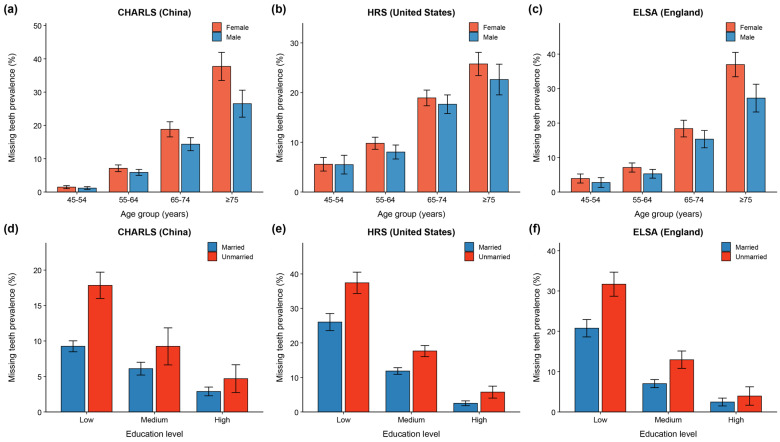
Baseline prevalence of missing teeth across sociodemographic subgroups in the three cohorts. Panels (**a**–**c**) show the prevalence of missing teeth by age group (45–54, 55–64, 65–74, and ≥75 years), stratified by sex, in CHARLS, HRS, and ELSA, respectively. Panels (**d**–**f**) show the prevalence of missing teeth by education level (low, medium, and high), stratified by marital status (married and unmarried), in CHARLS, HRS, and ELSA, respectively. Error bars indicate 95% confidence intervals.

The prevalence of baseline missing teeth across sociodemographic strata is shown in Figure 2. In all three cohorts, the prevalence of missing teeth increased markedly with age. In CHARLS, missing teeth were uncommon among participants aged 45–54 years, increased progressively in those aged 55–64 and 65–74 years, and rose sharply among those aged ≥75 years (Figure 2a). A similar age gradient was observed in HRS and ELSA, with the highest prevalence consistently seen in the oldest age group (Figure 2b,c). Within most age strata, women tended to have a higher prevalence of missing teeth than men.

Clear social gradients were also evident. In all three cohorts, participants with low educational attainment had the highest prevalence of missing teeth, whereas those with high education had the lowest prevalence (Figure 2d–f). In addition, unmarried participants consistently showed a higher prevalence of missing teeth than married participants across education strata. Together, these findings indicate that baseline missing teeth was more common among older and socially disadvantaged individuals.

### 3.3. Kaplan–Meier Analysis of Incident Cardiovascular Disease According to Missing Teeth Status

**Figure 3:**
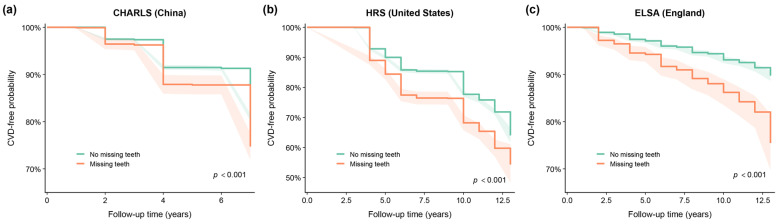
Kaplan–Meier curves for CVD-free survival according to baseline missing teeth status in the three cohorts. Panels (**a**–**c**) present CVD-free survival curves for participants with and without missing teeth in CHARLS, HRS, and ELSA, respectively. Green lines represent participants without missing teeth, and orange lines represent participants with missing teeth. Shaded areas indicate 95% confidence intervals. Group differences were assessed using the log-rank test. CVD, cardiovascular disease.

Kaplan–Meier curves for CVD-free survival according to baseline missing teeth status are presented in Figure 3. In all three cohorts, participants with missing teeth had a lower probability of remaining free of CVD over follow-up than those without missing teeth.

In CHARLS, the survival curves began to separate early and remained distinct throughout follow-up (Figure 3a). A similar pattern was observed in HRS, where the separation between the curves became more pronounced over time (Figure 3b). In ELSA, participants with missing teeth also exhibited persistently lower CVD-free survival during follow-up (Figure 3c). The log-rank test indicated statistically significant between-group differences in all three cohorts (all *p* < 0.001).

### 3.4. Association Between Missing Teeth and Incident Cardiovascular Disease

**Figure 4:**
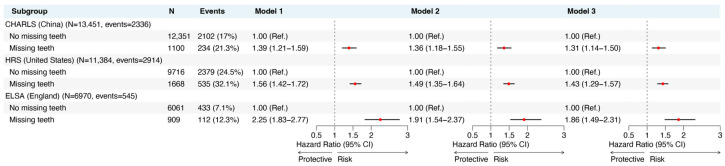
Association between baseline missing teeth and incident cardiovascular disease in CHARLS, HRS, and ELSA. Forest plots show hazard ratios (HRs) and 95% confidence intervals (CIs) from Cox proportional hazards models. Model 1 was unadjusted; Model 2 was adjusted for age, sex, education level, and marital status; and Model 3 was further adjusted for body mass index, smoking, drinking, hypertension, diabetes mellitus, cancer, chronic lung disease, and arthritis. Participants without missing teeth were used as the reference group. Red dots indicate point estimates, horizontal lines indicate 95% CIs, and the vertical dashed line indicates the null value (HR = 1).

The associations between baseline missing teeth and incident CVD estimated from Cox proportional hazards models are shown in Figure 4. Across all three cohorts, missing teeth were consistently associated with a higher risk of incident CVD.

In CHARLS, among 13,451 participants with 2336 incident events, the unadjusted model showed that participants with missing teeth had a significantly higher risk of incident CVD than those without missing teeth (HR, 1.39; 95% CI, 1.21–1.59). After adjustment for age, sex, education level, and marital status, the association remained significant (HR, 1.36; 95% CI, 1.18–1.55). Further adjustment for BMI, smoking, drinking, hypertension, diabetes mellitus, cancer, chronic lung disease, and arthritis attenuated the estimate slightly, but the association remained robust (HR, 1.31; 95% CI, 1.14–1.50). In HRS, missing teeth were likewise associated with a higher risk of incident CVD. The unadjusted HR was 1.56 (95% CI, 1.42–1.72), which decreased modestly after adjustment for demographic and socioeconomic factors (HR, 1.49; 95% CI, 1.35–1.64). In the fully adjusted model, participants with missing teeth still had a 43% higher risk of incident CVD than those without missing teeth (HR, 1.43; 95% CI, 1.29–1.57). In ELSA, the association was stronger in magnitude than in the other two cohorts. The unadjusted HR was 2.25 (95% CI, 1.83–2.77), and although attenuation was observed after sequential covariate adjustment, the fully adjusted model still showed a markedly elevated risk among participants with missing teeth (HR, 1.86; 95% CI, 1.49–2.31). Overall, the consistency of the fully adjusted associations across all three cohorts indicates that missing teeth were independently associated with subsequent CVD risk.

### 3.5. Subgroup Analyses

**Figure 5:**
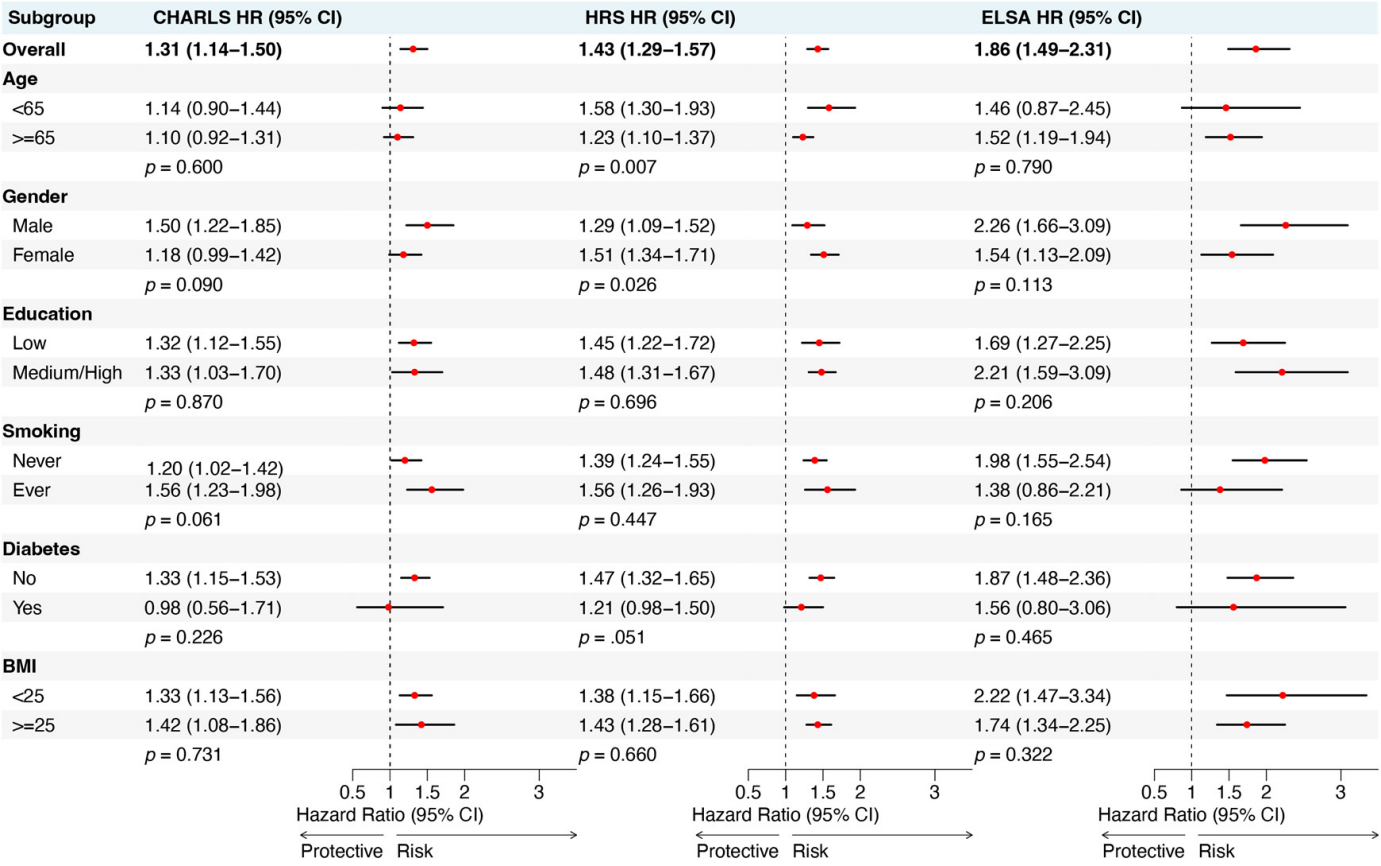
Subgroup analyses of the association between baseline missing teeth and incident cardiovascular disease. Forest plots show HRs and 95% CIs from fully adjusted Cox proportional hazards models across predefined subgroups stratified by age (<65 vs. ≥65 years), sex (male vs. female), education level (low vs. medium/high), smoking status (never vs. ever), diabetes mellitus (no vs. yes), and body mass index (<25 vs. ≥25 kg/m^2^). For each subgroup analysis, the stratification variable itself was excluded from the adjustment model. Interaction *p*-values were derived from likelihood ratio tests comparing models with and without the interaction term. Red dots indicate point estimates (HRs), horizontal lines indicate 95% CIs, and the vertical dashed line indicates the null value (HR = 1).

Subgroup analyses based on the fully adjusted model are shown in Figure 5. The association between missing teeth and incident CVD was examined across strata of age, sex, education level, smoking status, diabetes mellitus, and BMI.

Overall, the direction of the association was broadly consistent across subgroups in all three cohorts, with missing teeth generally associated with a higher risk of incident CVD in most strata. In CHARLS, no statistically significant interactions were observed, suggesting that the association was relatively stable across age, sex, education, smoking, diabetes, and BMI categories. In HRS, significant interactions were observed for age and sex, indicating some heterogeneity in the magnitude of association across these subgroups. In ELSA, although point estimates varied across subgroups, no statistically significant interactions were detected.

Taken together, these findings suggest that the positive association between missing teeth and incident CVD was broadly consistent across major demographic and clinical strata, with only limited evidence of heterogeneity.

### 3.6. Sensitivity Analyses

**Figure 6:**
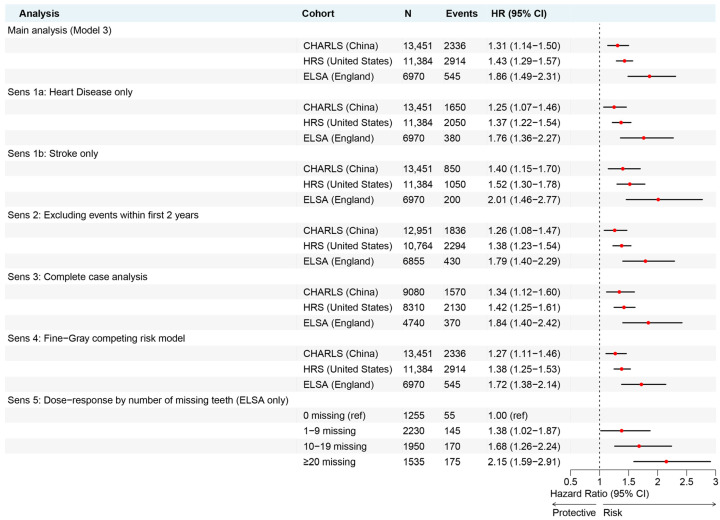
Sensitivity analyses for the association between baseline missing teeth and incident cardiovascular disease in CHARLS, HRS, and ELSA. Forest plots summarize the results of the primary fully adjusted model and prespecified sensitivity analyses, including restricting the outcome to heart disease only, restricting the outcome to stroke only, excluding events occurring within the first 2 years of follow-up, complete-case analysis, and Fine–Gray competing-risk models treating non-CVD death as a competing event. Estimates are presented as HRs or subdistribution HRs with 95% CIs. Red dots indicate point estimates, horizontal lines indicate 95% CIs, and the vertical dashed line indicates the null value (HR = 1).

Sensitivity analyses are presented in Figure 6. Across all three cohorts, the main findings remained materially unchanged under alternative analytic assumptions. When the outcome was restricted to heart disease only or stroke only, the association between missing teeth and incident CVD remained directionally consistent. Excluding participants who experienced CVD events within the first two years of follow-up did not substantially alter the results, arguing against substantial reverse causation. Complete-case analyses yielded estimates similar to those from the primary analyses, indicating that the results were not driven by the handling of missing covariate data. Likewise, Fine–Gray competing-risk models treating non-CVD death as a competing event produced results comparable to those of the primary analyses. When the outcome was restricted to heart disease only or stroke only, the association between missing teeth and incident CVD remained directionally consistent across cohorts, suggesting that the primary findings were not driven solely by one component of the composite outcome. Overall, these sensitivity analyses support the robustness of the primary findings.

Because ELSA provided information on the number of missing teeth, an additional sensitivity analysis was conducted in this cohort to evaluate whether more extensive tooth loss was associated with higher CVD risk. The results showed a graded increase in the risk of incident CVD across categories of tooth loss, suggesting that the association was stronger among participants with more severe tooth loss.

## 4. Discussion

In this longitudinal analysis of three nationally representative ageing cohorts, baseline missing teeth was consistently associated with a higher risk of incident cardiovascular disease, supporting its potential role as a simple marker of cardiovascular vulnerability in middle-aged and older adults. This association was observed across all three cohorts, remained significant after adjustment for a broad range of sociodemographic, behavioral, and clinical covariates, and was generally robust in subgroup and sensitivity analyses. In addition, participants with missing teeth had significantly lower CVD-free survival over follow-up in each cohort. Taken together, these findings support the hypothesis that missing teeth may serve as an accessible marker of elevated cardiovascular risk in ageing populations. The additional ELSA-specific analysis by the number of missing teeth further supports the clinical relevance of tooth-loss severity. Although the primary cross-cohort analysis used a binary definition of missing teeth to ensure comparability across CHARLS, HRS, and ELSA, the ELSA sensitivity analysis suggested a graded pattern, with more extensive tooth loss associated with higher risk of incident CVD. This finding suggests that more severe tooth loss may reflect greater cumulative oral disease burden, impaired masticatory function, poorer nutritional status, or broader systemic vulnerability.

An important consideration is that tooth loss may arise from heterogeneous causes, including periodontitis, dental caries, trauma, dental treatment decisions, limited access to dental care, and financial barriers. These causes may have different implications for cardiovascular risk. In particular, tooth loss resulting from periodontitis may reflect long-standing oral infection and chronic inflammatory burden, whereas tooth loss due to trauma may not carry the same biological meaning. Therefore, in the present study, missing teeth should be interpreted as a broad marker of cumulative oral health impairment rather than a specific indicator of periodontal disease.

Our findings are broadly consistent with previous epidemiological evidence. A dose–response meta-analysis of prospective cohort studies reported that tooth loss was associated with increased risks of CVD and stroke [7]. This interpretation is supported by the Scottish Health Survey, in which edentulousness was associated with higher CVD mortality [8], and by a large Korean cohort study showing that poorer oral health, including tooth loss, was associated with subsequent coronary heart disease [9]. Additional meta-analytic evidence has further suggested that greater tooth loss is associated with adverse atherosclerotic cardiovascular and mortality outcomes [10,11]. Together with the present findings, these studies support the view that tooth loss may be a meaningful marker of cardiovascular risk.

Several biological and behavioral pathways may plausibly explain the observed association. Tooth loss is often the consequence of long-standing oral disease, especially periodontitis, which has been linked to systemic inflammation, endothelial dysfunction, and atherogenesis [3,4,5]. Chronic oral inflammation may promote vascular injury through circulating inflammatory mediators, microbial translocation, and immune activation [4,5]. In addition, tooth loss may impair mastication and contribute to poorer dietary quality, including lower intake of fruits, vegetables, and other cardioprotective nutrients [6]. Therefore, missing teeth may reflect both long-term inflammatory burden and reduced oral function, each of which may be relevant to the development of CVD.

Our findings are also compatible with the broader literature on periodontal disease and cardiovascular risk. A systematic review and meta-analysis of longitudinal studies reported that people with periodontal disease had a higher risk of incident CVD than those without periodontal disease [20]. This broader oral-systemic evidence strengthens the interpretation that missing teeth may capture a clinically relevant dimension of long-term oral disease burden related to vascular risk.

The cross-national nature of the present study is an important strength. Most previous studies have been conducted in single populations, whereas our analysis applied a harmonized framework to three nationally representative ageing cohorts from China, the United States, and England [12,13,14]. Despite differences in healthcare systems, oral health service use, population structure, and baseline cardiovascular risk, the direction of association was highly consistent across cohorts. Although the magnitude of association was stronger in ELSA than in CHARLS and HRS, the overall pattern remained remarkably similar. This cross-national consistency supports the potential generalizability of the observed relationship.

In cohort-specific analyses, the association was present in all three populations but differed in magnitude. In CHARLS, missing teeth were associated with a 31% higher risk of incident CVD after full adjustment. In HRS, the corresponding excess risk was 43%, while in ELSA the association was stronger, with an 86% higher risk. The consistency in direction across China, the United States, and England supports the robustness and potential generalizability of the association. However, the stronger estimate in ELSA should be interpreted cautiously, as it may reflect differences in age distribution, oral health profiles, healthcare access, outcome ascertainment, follow-up structure, event numbers, or residual confounding.

The three cohorts differed substantially in demographic, behavioral, and clinical characteristics. These differences likely reflect variation in population age structure, socioeconomic conditions, smoking and drinking patterns, obesity and cardiometabolic risk profiles, oral healthcare access, and national healthcare systems. Such heterogeneity may partly explain the different magnitudes of association observed across cohorts. For example, differences in baseline CVD risk, competing risks, oral health service use, and the clinical meaning of reported tooth loss may influence the estimated hazard ratios. Nevertheless, the association between missing teeth and incident CVD was consistently positive in all three cohorts, suggesting that the relationship is not restricted to a single country or healthcare setting.

The interplay between diabetes, periodontitis, and CVD deserves particular attention. Periodontitis is more common and often more severe among individuals with diabetes, and chronic periodontal inflammation may further contribute to systemic inflammatory activation, endothelial dysfunction, and cardiovascular risk. In our study, diabetes mellitus was adjusted for in the fully adjusted model, and subgroup analyses by diabetes status showed broadly consistent associations. Nevertheless, because harmonized data on clinically diagnosed periodontitis were unavailable, we could not determine whether the observed association differed according to periodontal disease status or whether periodontitis mediated part of the association between missing teeth and CVD.

At the same time, caution is needed in causal interpretation. Observational associations between oral health and CVD can be influenced by shared determinants, including smoking, socioeconomic disadvantage, diabetes, and access to healthcare. Although our analyses adjusted for a broad range of such covariates and the findings remained stable in multiple sensitivity analyses, residual confounding cannot be excluded. A recent quasi-experimental study using exogenous variation in childhood water fluoridation reported evidence supporting a causal effect of tooth loss on cardiovascular diseases in U.S. adults [21]. While this does not establish causality in the present study, it suggests that the relationship may not be entirely attributable to confounding alone.

From a clinical and public health perspective, our findings are relevant because missing teeth are easy to identify in both clinical and community settings and may help flag individuals at increased cardiovascular risk. Recent reviews have argued that oral health should be considered a potentially modifiable component of cardiovascular disease prevention [22], and an umbrella review concluded that higher-level evidence supports an association between periodontal disease, tooth loss, and cardiovascular diseases, although methodological limitations remain [23]. Our results add longitudinal, cross-national evidence to this literature and support greater integration of oral health into healthy ageing and cardiovascular prevention frameworks.

Several limitations should be acknowledged. First, as in all observational studies, residual confounding cannot be excluded. Second, missing teeth were analyzed as a dichotomous exposure, which precluded assessment of dose–response associations according to the number or distribution of missing teeth. Third, detailed oral clinical measures, such as periodontal probing depth, denture use, chewing function, and oral hygiene behavior, were not uniformly available across the three cohorts. Fourth, exposure and outcome ascertainment relied at least partly on self-report, which may have introduced misclassification. Finally, cross-cohort differences in measurement and follow-up structure may also have contributed to variation in the magnitude of the estimated associations.

Future studies should examine whether the extent, location, timing, and progression of tooth loss are differentially associated with specific cardiovascular outcomes. Studies incorporating periodontal examination, inflammatory biomarkers, oral microbiome data, and dietary mediators would help clarify the underlying mechanisms. Interventional and quasi-experimental studies are also needed to determine whether preventing tooth loss or improving oral function can reduce future cardiovascular risk.

## 5. Conclusions

In this longitudinal analysis of three nationally representative cohorts from China, the United States, and England, a baseline of missing teeth was consistently associated with a higher risk of incident cardiovascular disease among middle-aged and older adults. This association remained significant after adjustment for a wide range of sociodemographic, lifestyle, and clinical factors and was generally robust across subgroup and sensitivity analyses. These findings suggest that missing teeth may serve as a simple and clinically accessible marker of elevated cardiovascular risk in ageing populations and support greater integration of oral health into cardiovascular prevention and risk assessment strategies.

## Figures and Tables

**Table 1:** Baseline Characteristics of Study Participants by Database.

Characteristics	Level	Overall (*n* = 31,805)	CHARLS (*n* = 13,451)	HRS (*n* = 11,384)	ELSA (*n* = 6970)	*p*	SMD
Age, years		61.00 [54.00, 69.00]	57.00 [51.00, 64.00]	65.00 [57.00, 72.00]	62.00 [56.00, 71.00]	<0.001	0.375
Gender	Female	17,861 (56.2)	6788 (50.5)	7081 (62.2)	3992 (57.3)	<0.001	0.123
	Male	13,944 (43.8)	6663 (49.5)	4303 (37.8)	2978 (42.7)		
Education level	Low	11,449 (36.0)	7016 (52.2)	2148 (18.9)	2285 (32.8)	<0.001	0.452
	Medium	13,189 (41.5)	3158 (23.5)	6596 (57.9)	3435 (49.3)		
	High	7167 (22.5)	3277 (24.4)	2640 (23.2)	1250 (17.9)		
Marital status	Divorced	3445 (10.8)	1142 (8.5)	1537 (13.5)	766 (11.0)	<0.001	0.226
	Married	23,217 (73.0)	10,898 (81.0)	7507 (65.9)	4812 (69.0)		
	Single	930 (2.9)	117 (0.9)	421 (3.7)	392 (5.6)		
	Widowed	4213 (13.2)	1294 (9.6)	1919 (16.9)	1000 (14.3)		
Body mass index, kg/m^2^		25.39 [22.39, 28.80]	23.01 [20.74, 25.56]	27.30 [24.20, 30.90]	27.35 [24.70, 30.57]	<0.001	0.565
Current smoking	No	24,931 (78.4)	9195 (68.4)	9761 (85.7)	5975 (85.7)	<0.001	0.243
	Yes	6874 (21.6)	4256 (31.6)	1623 (14.3)	995 (14.3)		
Current drinking	No	14,713 (46.3)	8739 (65.0)	5262 (46.2)	712 (10.2)	<0.001	0.648
	Yes	17,092 (53.7)	4712 (35.0)	6122 (53.8)	6258 (89.8)		
Missing teeth	No	28,128 (88.4)	12,351 (91.8)	9716 (85.3)	6061 (87.0)	<0.001	0.110
	Yes	3677 (11.6)	1100 (8.2)	1668 (14.7)	909 (13.0)		
Hypertension	No	20,820 (65.5)	10,565 (78.5)	5977 (52.5)	4278 (61.4)	<0.001	0.296
	Yes	10,985 (34.5)	2886 (21.5)	5407 (47.5)	2692 (38.6)		
Diabetes mellitus	No	28,957 (91.0)	12,804 (95.2)	9696 (85.2)	6457 (92.6)	<0.001	0.182
	Yes	2848 (9.0)	647 (4.8)	1688 (14.8)	513 (7.4)		
Cancer	No	29,945 (94.2)	13,353 (99.3)	10,101 (88.7)	6491 (93.1)	<0.001	0.244
	Yes	1860 (5.8)	98 (0.7)	1283 (11.3)	479 (6.9)		
Chronic lung disease	No	29,731 (93.5)	12,311 (91.5)	10,779 (94.7)	6641 (95.3)	<0.001	0.085
	Yes	2074 (6.5)	1140 (8.5)	605 (5.3)	329 (4.7)		
Arthritis	No	19,485 (61.3)	9274 (68.9)	5510 (48.4)	4701 (67.4)	<0.001	0.234
	Yes	12,320 (38.7)	4177 (31.1)	5874 (51.6)	2269 (32.6)		
Residence (%)	Rural	11,982 (48.2)	8430 (62.7)	3552 (31.2)	-	<0.001	0.437
	Urban	12,853 (51.8)	5021 (37.3)	7832 (68.8)	-		

Note: Data are presented as mean (SD), median [IQR], or *n* (%). Residence data is not available for ELSA. *p* values from Kruskal–Wallis test (continuous) or Chi-squared test (categorical) across the three databases. SMD = Standardized Mean Difference. CHARLS = China Health and Retirement Longitudinal Study; HRS = Health and Retirement Study; ELSA = English Longitudinal Study of Ageing.

## Data Availability

The datasets analyzed in this study are publicly available from the official websites of the China Health and Retirement Longitudinal Study (CHARLS), the Health and Retirement Study (HRS), and the English Longitudinal Study of Ageing (ELSA), subject to registration and approval by the corresponding data custodians. No new data were generated in this study. The analytic code is available from the corresponding author upon reasonable request.

## Abbreviations

The following abbreviations are used in this manuscript:

BMI	Body mass index
CHARLS	China Health and Retirement Longitudinal Study
CI	Confidence interval
CVD	Cardiovascular disease
ELSA	English Longitudinal Study of Ageing
HR	Hazard ratio
HRS	Health and Retirement Study
IRB	Institutional Review Board
IQR	Interquartile range
KM	Kaplan–Meier
SMD	Standardized mean difference
